# EEG-Based BCI System Using Adaptive Features Extraction and Classification Procedures

**DOI:** 10.1155/2016/4562601

**Published:** 2016-08-17

**Authors:** Valeria Mondini, Anna Lisa Mangia, Angelo Cappello

**Affiliations:** Department of Electrical, Electronic and Information Engineering (DEI), University of Bologna, Viale del Risorgimento, 40136 Bologna, Italy

## Abstract

Motor imagery is a common control strategy in EEG-based brain-computer interfaces (BCIs). However, voluntary control of sensorimotor (SMR) rhythms by imagining a movement can be skilful and unintuitive and usually requires a varying amount of user training. To boost the training process, a whole class of BCI systems have been proposed, providing feedback as early as possible while continuously adapting the underlying classifier model. The present work describes a cue-paced, EEG-based BCI system using motor imagery that falls within the category of the previously mentioned ones. Specifically, our adaptive strategy includes a simple scheme based on a common spatial pattern (CSP) method and support vector machine (SVM) classification. The system's efficacy was proved by online testing on 10 healthy participants. In addition, we suggest some features we implemented to improve a system's “flexibility” and “customizability,” namely, (i) a flexible training session, (ii) an unbalancing in the training conditions, and (iii) the use of adaptive thresholds when giving feedback.

## 1. Introduction 

A brain-computer interface (BCI) is a system creating a direct communication channel between the brain and the outside, bypassing the ordinary outputs of the central nervous system like peripheral nerves and muscles [1]. Noninvasive BCIs based on electroencephalography (EEG) are the most widespread systems [2], and motor imagery (MI) is one of the most commonly used control strategies. Since the imagination of a voluntary movement induces specific patterns of ERD (event-related desynchronization) and ERS (event-related synchronization) in the EEG mu (8–13 Hz) and beta (13–30 Hz) rhythms over the sensorimotor cortex, different MIs generate different EEG spatial patterns [3], which can be classified and used for communication or control purposes.

However, voluntary control of sensorimotor rhythms (SMR) by imagining a movement is a skilful unintuitive task [2], so a varying amount of user training is usually required [4]. The typical BCI training approach includes the steps: (1) preliminary data acquisition in a cue-guided paradigm without feedback, (2) setup of a subject-specific classifier based on the acquired data, and (3) online BCI operation with feedback based on the previously trained classifier. Since feedback plays a key role in making the user learn how to produce the correct EEG modulations, the system should start giving feedback as soon as possible to boost the training process [5]. However, given the nonstationary nature of EEG and, specifically, the effect of feedback training which modifies the user's EEG patterns with respect to nonfeedback data used for calibration [6], an adaptation of the system parameters might be useful to improve the feedback quality and speed up the training process. Over the past decade, several adaptive systems have been proposed to explore both these aspects (providing feedback as early as possible while continuously adapting the underlying classifier model, promoting a coadaptation of both user and machine), namely, [5, 7–16].

Vidaurre et al. and Faller et al. [5, 7–11] described several online adaptive systems employing adaptive autoregressive (AAR) parameters and/or logarithmic band power features, combined with quadratic discriminant analysis (QDA) or linear discriminant analysis (LDA) classifiers. Despite methodological differences, all the systems were fully automated, gave feedback from the very first moment [7–9] or at least after a few minutes of calibration [5, 10, 11], and updated the classifier's parameters trialwise, using the most separable time-segment of the previous trial for adaptation. All the systems were tested online on healthy [5, 7–9] or physically impaired [10, 11] users, showing peak online accuracies that tended to increase in just two to three days. In the works [12, 13] Xia et al. and Qin et al. presented two different adaptive methods, involving both common spatial pattern (CSP) [17] filtering and linear support vector machine (SVM) classifier [18, 19]. Both methods aimed at improving the user's training process, showing their efficacy either in online experiments [12] or in simulations of available datasets [13]. In [14, 15] Vidaurre et al. introduced a higher coverage setup (48 electrodes) and more elaborate adaptive pattern, targeting people who could not previously achieve BCI control (the BCI “illiterates” [20]). Finally, [16] also inserted an unsupervised adaptation scheme.

Even though the results of these studies are already valuable, we suggest that other aspects could also be taken into account to improve the training process further. Beyond the developed adaptive algorithm, the way the system interacts with the user is also important. Since BCI performance greatly varies across users [20], we suggest that “short calibration,” “automaticity,” and “adaptivity” should be flanked by “flexibility,” another key feature to improve the BCI training process. Specifically we propose to

- adapt the training session depending on the user's ability (i.e., the session should not be needlessly long if the user reaches enough control, whereas training could be restarted from the beginning if the system keeps performing at chance level for too long, giving the user the possibility to try a different imagination strategy);
- try to keep challenging the user independently from his/her performance (e.g., giving feedback only in case the distance from the decision boundary exceeds an adaptive threshold);
- present the training conditions (e.g., left/right hand motor imagery) not in equal numbers, but to bias in favour of the condition which is currently the hardest to predict.

We therefore introduce here a system designed to incorporate all these aspects thanks to its modular structure. The system is a cue-paced BCI using MI of left versus right hand to control the flexion-extension of a 1 DOF-modelled arm on a screen, including a simple adaptive scheme based on CSP filtering and SVM classification [12]. The implemented adaptive scheme is similar to some previously proposed algorithms [21–24], generally defined as ACSP (adaptive common spatial pattern): we did not include these works among the previously cited ones [5, 7–16] because they have a different aim, that is, generically dealing with EEG inter- and intrasubject nonstationarities, rather than improving the user training process. However, beyond the implemented adaptive strategy, the system we describe was conceived as a whole, from training phase to utilization, and therefore includes a short calibration module without feedback (less than 3 minutes), followed by several repetitions of an adaptive module with feedback. Finally, as the user proves skilled enough to control the flexion-extension of the simulated arm, adaptation ends and the system switches to a module where the simulated arm is used to reach targets on the screen. The system was tested online on 10 healthy participants for three days each.

## 2. Materials and Methods 

### 2.1. Signal Acquisition and Preprocessing

**Figure 1:**
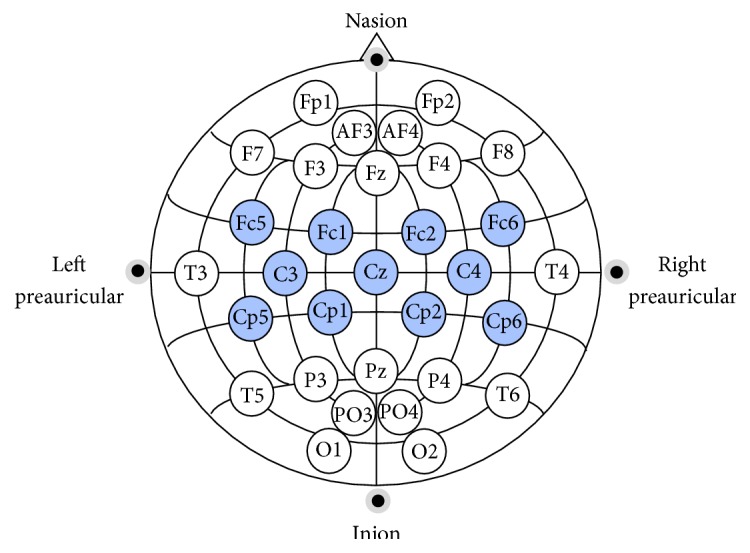
The 11 recorded channels.

The EEG signals were acquired using a Brainbox EEG-1166 amplifier with 128 Hz sample frequency. Eleven passive wet Ag/AgCl electrodes were used over the sensorimotor areas (Figure 1), together with a reference electrode on the right ear lobe and ground electrode on the forehead.

As suggested in [25], all signals were rereferenced with common average reference (CAR). Since the right ear potential was included in the averaging operation, the 11 brain signals were kept linearly independent. After rereferencing, signals were filtered in the 8–30 Hz band, as suggested in [26].

For feature extraction, the common spatial pattern (CSP) algorithm [17] was used. As is known, this algorithm finds the matrix** W** that maps the EEG multichannel data in a space where the difference in variance between the 2 classes is maximized [27]. Given** X** the Nxt matrix of recorded and preprocessed signals (*N* channels acquired; *t* number of samples), the matrix of new time-series in** Z** (Nxt) is therefore obtained as **Z** = **W** · **X**. To compute the** W** matrix, the CSP method considers the simultaneous diagonalization of the averaged normalized covariance matrices of the 2 classes (right/left hand MI). Further details on the CSP algorithm can be found in [28].

To compose the feature vector** f**, we considered the log-transformed normalized variances of the time-series in the first and last 2 rows of** Z**, as suggested in [26]. The feature vectors** f** were later used to train a support vector machine (SVM) classifier [18, 19], with a linear kernel and a soft margin equal to 1.

### 2.2. The Online BCI System: The Three Modules

This section outlines the 3 modules in our system:* Training* (*T*),* Training & Updating* (*U*), and* Classification* (*C*). The 3 modules have different functions and were designed to be assembled together to set up a typical training session. The entire system was developed using LabVIEW. An overview of the main features and the differences between the modules is given in Table 1.

#### 2.2.1. Training (*T*)

*T* module makes a first estimation of the** W** matrix and the SVM parameters, without any feedback for the user.

**Figure 2:**
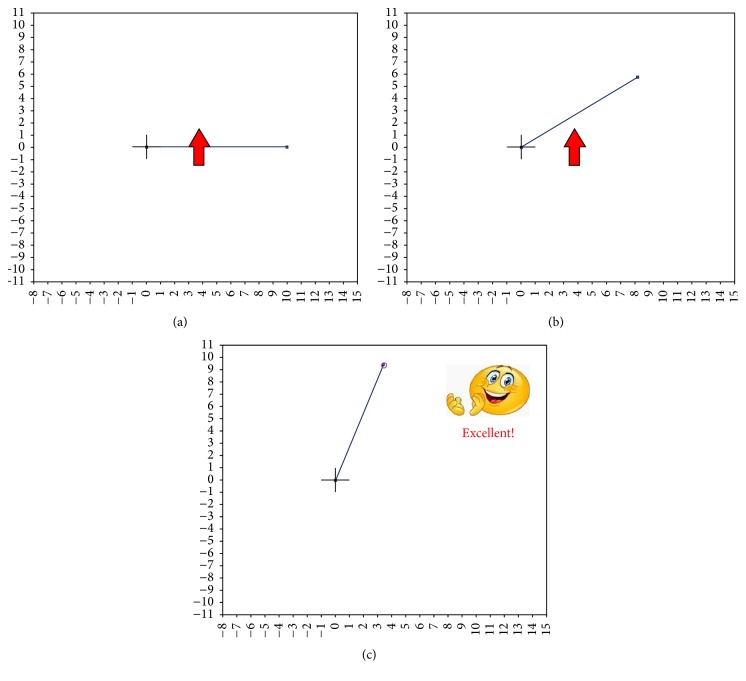
Upward pointing arrow in the *T* module (a) and in the *U* module (b). On (c), a target reached in *C* module is shown.

*MI Instructions and Feedback.* When *T* starts, an upward/downward pointing arrow appears on the screen over the modelled arm (Figure 2(a)). Depending on the direction of the arrow (upward or downward), the user is requested to imagine the movement of his right or left hand, respectively. No feedback is given to the user.

*Arrow Balancing*. The arrow is presented in all 14 times (7 upwards and 7 downwards); each time it is visible for 10 s, with 2.5 s of rest. The training process without feedback therefore lasts less than 3 minutes. The arrow presentation order is randomized.

**Figure 3:**
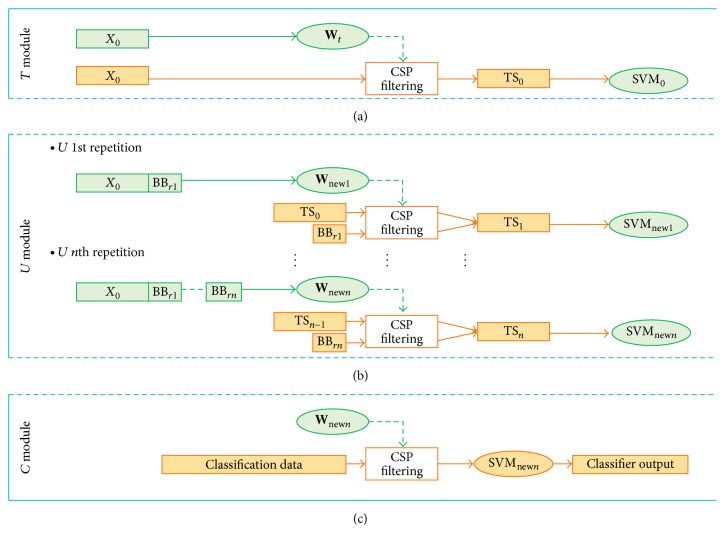
A conceptual schema describing the evaluation/use of **W** matrix and SVM classifier in the three modules. (a) The first setup of **W** and SVM, starting from the time-domain EEG signal (*X*_0_), in *T* module. (b) A diagram describes how **W** and SVM are updated in *U* module, starting from the stored signal portions, the current BB list, and the previous training set. (c) The schema shows how the definitive **W** and SVM are used in *C* module to classify the incoming signals and provide the feedback.

**W** *and SVM First Computation.* When the arrow is visible, 2 s-long portions of EEG signal are extracted every 0.5 s (only the first portion is extracted after 2 s) so that 17 portions are extracted from every arrow repetition for a total of 238 (17 × 14) signal portions. The 238 portions are labelled according to the corresponding arrow's direction and used to (i) estimate** W**, (ii) extract the feature vector** f**, and (iii) train the SVM classifier (Figure 3(a)). At the end of *T* module, the software automatically switches to *U*.

#### 2.2.2. Training & Updating (*U*)

*U* module is designed to be reiterated several times (*U* repetition). The main purpose of *U* is to guide the user training by providing feedback while adapting the system's parameters. Both** W** matrix and the SVM classifier are adapted at the end of each *U* repetition, after making a selection over the recorded signals. This module also introduces the concepts of adaptive thresholds and unbalancing in the training conditions.

*MI Instructions and Feedback*. Similarly to *T* module, MI instructions are given by presenting an upward/downward pointing arrow over the modelled arm; each arrow is visible for 10 s with 2.5 s rest, and 2 s-long EEG signal portions are extracted every 0.5 s. Nevertheless, in *U* module a time-discrete feedback, encoded in a 5° increase/decrease of the model's shoulder angle, is added according to the classifier's output (Figure 2(b)). Specifically, after filtering each new portion with** W**, the feature vector** f** is extracted and the output of the SVM classifier is used to give feedback to the user.

*Adaptive Thresholds.* To keep challenging the user, we decided to provide feedback only if the analysed EEG signal portions were “distant enough” from the classifier's separation hyperplane. Since the user's ability to produce different MI could be imbalanced, we considered 2 independent thresholds for right and left hand MI. Both start from 0 and are continuously adapted as 60% of the average of the right/left hand imagery feature's distances obtained from the start of the session. For threshold computation, only the “correct” features (i.e., when the classifier's output agrees with the arrow direction) are taken into account. The choice of 60% was based on preliminary experiments (we tried to choose a value able to challenge the participants without discouraging them).

**W** *and SVM Use and Updating*. In each *U* repetition, the arrow is shown in all 10 times, and each time it is visible for 10 s. Thus, at the end of *U*, 170 new signal portions (17 signal portions from each arrow × 10 arrow repetitions) have been processed and are theoretically available to update** W** and the classifier. Out of the total 170 portions, we decided to keep in the memory only the ones correctly classified and above the threshold. The resulting list is called the best portions list (B list). Later, the B list is further reduced, equalizing the number of right hand and left hand features. In this balancing operation, the B elements with the shortest distance from the hyperplane are removed first. After this operation, we obtain a list of the best balanced portions (BB list).

The BB list is first used to update** W** (**W**_new_) by extracting the covariance matrices from the BB signals. Specifically, **W**_new_ is computed averaging the new BB covariance matrices together with all the matrices selected from the start of the session (i.e., those from *T* module and all those obtained from BB lists in each *U* repetition completed up to that moment). In this way, we aimed to gradually stabilize the** W** matrix, since it is the result of the averaging of an increasing number of covariance matrices.

Once **W**_new_ is computed, the BB list is also used to update the classifier's training set. First of all, the old training set must be remapped according to **W**_new_. Once the training set has been remapped, the BBs are also transformed with **W**_new_, and the new features obtained are used to* replace* the older ones in the training set. We opted for* replacement* instead of simply* adding* the new features to the training to avoid an increment of computational weight. Once the training set has been updated, the new SVM classifier (SVM_new_) can be retrained.

To further clarify the updating procedure, the steps made at the end of each *U* repetition to update the system's parameters are shown in Figure 3(b) and summarized here.

(1) At the end of *U*, only the B are kept in the memory. The list is further reduced balancing the samples of the 2 classes (right hand and left hand imagery), thus obtaining the BB list.
(2) New normalized covariance matrices are extracted from the BB signals. For each class, the new matrices are averaged along with the previous ones. **W**_new_ is therefore computed.
(3) The old classifier's training set is remapped with **W**_new_.
(4) New features are extracted from BB according to **W**_new_. These new features are used to replace the older ones in the classifier's training set. SVM_new_ can be set up.
(5) Repeat *U* or switch to *C* module.

*Arrow Imbalancing*. The last characteristic of *U* module is the imbalance in the presentation of the arrows. In particular, to maximize the probability of updating the classifier (given the balancing operation from B to BB) and to customize the user's training with a stronger stimulation of the most critical MI condition, the pointing arrow corresponding to the most misclassified task is presented more frequently. To clarify, at the end of each *U* repetition, the number of misclassifications for each class is counted, and the ratio between these two numbers is computed. Depending on this ratio, the arrow directions in the following *U* repetition can be imbalanced up to 7 : 3 (or 3 : 7), in favour of the previously most misclassified class. As the user improves his/her skills in both MI conditions, the ratio between the arrow directions will tend to return to a balanced 5 : 5.

*Accuracy.* At the end of each *U* repetition, the classification accuracy of the current step is evaluated as the ratio between the correctly classified features and the total number of processed features. Because of the imbalance in favour of the most misclassified class, the classification accuracy obtained is underestimated. As soon as the classification accuracies are stable and good enough (see Section 2.3.2), the system automatically switches to *C* module.

#### 2.2.3. Classification (*C*)

*C* module is designed to test the user's ability to control the flexion-extension of the modelled arm to reach targets on the screen. In *C* module the adaptive thresholds,** W**, and the SVM classifier are no longer updated.

*MI Instructions and Feedback.* In *C* module, the MI instruction is no longer by presentation of the pointing arrow, but through the appearance of a ball-shaped target (Figure 3(c)). The user has to reach the target with the arm's end-point as soon as possible, with a timeout of 120 s. As for *T* and *U*, 2 s-long EEG signal portions are extracted and classified every 0.5 s. Every *C* repetition consists of 5 targets presented in succession on the screen, with a 5 s pause when the target is reached. As the user reaches the target, a smiling face appears on the screen (Figure 2(c)). Otherwise, a sad face is shown if the timeout expires.

*Adaptive Thresholds. *As in *U* module, in *C* the feedback (the modelled arm's movement) is given only if the extracted features are above threshold. However, in *C* phase the 2 thresholds are no longer updated, so the thresholds computed in the last repetition of *U* are used.

**W** *and SVM Use*.** W** and the SVM classifier are no longer updated. Thus,** W** and SVM computed in the last repetition of *U* are used.

*Accuracy*. At the end of *C* the classification accuracy can be estimated as the ratio between the correctly classified features and the total number processed. Since the participant is asked to reach the target as quickly as possible, the “correct” label can be derived depending on the target's position. Specifically, the classification accuracy is computed considering (i) the targets reached; (ii) the first 30 s of “timeout” cases. This assumption was made because after a while participants tended to give up trying to reach the target and simply waited for the timeout, thereby invalidating the deduction of the “correct” label.

### 2.3. Experimental Setup

#### 2.3.1. Participants and Setup

The described system was tested on 10 healthy volunteers (P01–P10). Eight of them had no previous MI experience, while P01 and P04 had totalled up 461 and 437 minutes of MI experience, respectively, in the same year as the experiment. The participants, 7 females and 3 males, were all right-handed (according to Edinburgh inventory [29]) and were aged 26.5 ± 2 years (mean ± standard deviation). All volunteers were thoroughly informed beforehand of the nature and specifics of the experiments, and all of them gave written, informed consent.

During the experiment, the participants sat in front of the PC screen with their arms relaxed and in a comfortable position. To avoid EEG artefacts, the participants were asked not to contract facial muscles and to keep their gaze fixed during the trials. The system did not include any online artefact rejection algorithm. However, to check the absence of systematically occurring artefacts, an experienced inspector examined the acquired signals after each training session. In case a systematic artifactual activation was found, the entire session was excluded from results.

#### 2.3.2. Experimental Paradigm: The “Flexible” Training Session

Each participant took part in 6 training sessions (2 sessions per day). However, to fully customize the training process, we adapted the type and length of each training session depending on the participant's performances.

First of all, each training session was composed of 1 initial *T* and a maximum of 16 *U* repetitions. However, if the average classification accuracy in the last 3 repetitions of *U* was below 40%, *T* module was automatically repeated to reset the system's parameters (and to give the participant an opportunity to try a different imagination strategy). After the reset, the participant could complete the remaining repetitions of *U* module.

On the other hand, if participants proved skilled enough, they had the possibility to finish the session ahead of time. In particular, as soon as the average classification accuracy in the last 6 repetitions of *U* was above the criterion level of 70%, the participant's performances were considered good and stable enough and the system automatically switched to *C* module. Every time *C* phase was reached, the participant performed 3 repetitions of *C* and the session ended. Otherwise, the session simply concluded after the 16 repetitions of *U* module.

**Figure 4:**
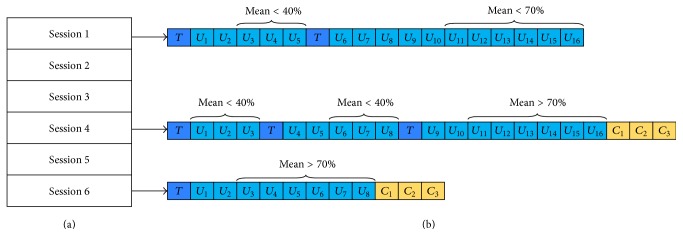
In this work, each participant took part in a total of 6 sessions (a). (b) shows 3 possible compositions of the session. In the top example, *T* module is repeated because the average accuracy in the previous 3 *U* repetitions was lower than 40%. At the end of the session, *C* phase is not even reached because the average accuracy in the last 6 repetitions was <70%. The middle example is similar to the top one (*T* module is indeed repeated), but this time *C* phase is reached at the end of the session. Finally, the bottom example shows the case of a shorter session.

To clarify, 3 examples of possible compositions of a session, according to the experimental paradigm, are given in Figure 4.

### 2.4. System Evaluation

#### 2.4.1. Accuracy

As previously emphasized by Billinger et al. in [30], a consequence of the increasing interest in BCI research is that papers tend to routinely highlight results and methods that improve accuracy or reduce illiteracy with respect to earlier work. The problem is that different (and barely comparable) methods of evaluation are often used, the procedures are not described in sufficient detail, and the value of chance level (i.e., the expected best performance obtainable by chance alone [30]) is not reported for comparison. However, showing classification results alone is often not enough, and even accuracies as high as 90% can be meaningless if classes are imbalanced or there are too few trials [30].

The present work reports the average classification accuracy together with its chance level *p*_0_. Since the arrow presentation was generally imbalanced, *p*_0_ was evaluated without loss of generality from confusion matrices, as described in [30]. We also evaluated the significance (*α* = 0.05) of the difference between mean accuracy and chance level using confidence intervals [30].

We briefly report here the computation of *p*_0_, as in [30]. Considering the confusion matrices, 
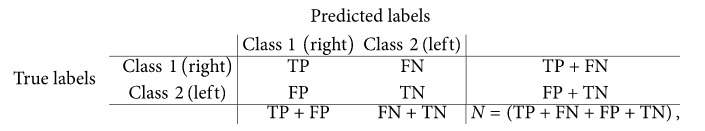
(1)where TP is the number of “true positive” classified signal portions, FN is the “false negative” ones, FP is the “false positive” ones, TN is the “true negative” ones, and* N* = (TP + FN + FP + TN) is the total number of classified signal portions of the session. Given the definition of *p*_0_ from [30]

(2) $$\begin{array}{c} p_{0}=\frac{\sum C_{i,\text{:}}C_{\text{:},i}}{N^{2}}, \end{array}$$

*p*_0_ is computed as

(3) $$\begin{array}{c} p_{0}=\frac{\left(TP+FN\right)\left(TP+FP\right)+\left(FP+TN\right)\left(FN+TN\right)}{{\left(TP+FN+FP+TN\right)}^{2}}. \end{array}$$

As regards the significance of the difference between average accuracy and chance level *p*_0_ using confidence intervals, we computed the lower bound of the confidence interval as in [30]

(4) $$\begin{array}{c} p_{l}=\hat{p}-z_{1-\alpha /2}\sqrt{\frac{\hat{p}\left(1-\hat{p}\right)}{N+4}}, \end{array}$$

where *z*_1−*α*/2_ is the 1 − *α*/2 quantile of the standard normal distribution and

(5) $$\begin{array}{c} \hat{p}=\frac{\left(TP+TN\right)+2}{N+4} \end{array}$$

is the adjusted average classification accuracy. If *p*_0_ > *p*_*l*_, the average classification accuracy cannot be considered significantly better than chance [30].

In our opinion, the average accuracy is a representative estimation of the user's real ability to control the system. However, since some of the previous works [5, 7, 8] extensively report only peak accuracies (in [5] the average accuracy curves are also displayed, but they regard only the last training session), we also add the information on peak accuracies to allow for comparability. Peak accuracy is obtained by computing the average classification accuracy of every time-point of the trial and reporting the peak value [5, 7, 8]. Finally, to be complete, we also report the values of information transfer rate (ITR) [31].

As explained in Section 2.3.2, *T* module can be repeated in a session if performances are too low. This option was introduced to give users the possibility to try different strategies and avoid annoying them with discouraging feedback. If *T* module was repeated, we considered for evaluation of accuracies chance level only the repetitions of *U* following the last *T*. The average accuracy and its chance level are also reported for *C* if it was reached.

All the accuracies shown in this paper reflect the obtained online accuracies, without rejection of artefactual trials. To be complete, in a posterior analysis an experienced inspector visually checked the EEG time-courses to reject artifactual data and recompute the accuracies. During this analysis, the inspector was blinded to the contents of the trials. Since the average rejection rates were overall reasonably low (8.4 ± 4.5% of artifactual trials, mean ± standard deviation) and the recomputed accuracies were not significantly different from the ones without artefact rejection, we decided to only report the values of real obtained online accuracies, without artefact rejection.

To evaluate the improvements in participants' performances, we tested the significance of the difference in both peak and average accuracy between the first and the last session.

#### 2.4.2. Time Effect

The system described here is a cue-paced BCI. However, going in the direction of asynchronous BCIs, we think that it is important for a system to classify each time-point equally well, since the BCI should recognize the mental state whenever it occurs. To test this ability of the system, we computed the average accuracy curves of each time-point of the trial in the last session, as in [5].

#### 2.4.3. Efficacy of the Adaptive Thresholds

As previously introduced, to keep challenging the users encouraging them to produce increasingly clear mental states, we decided to give feedback only if the produced EEG pattern exceeded an adaptive threshold from the decision boundary. However, to test if the “above threshold” signals were actually the most representative of the 2 classes and to verify that the patterns produced actually resembled the physiological ERD/ERS MI patterns, we decided to compute *r* ^2^ maps for each participant and session to compare the signals in the conditions: (i) correct classification and above threshold and (ii) only correct classification irrespective of the threshold.

The coefficient of determination *r* ^2^ is a commonly used (e.g., [15, 25, 31–33]) index in the BCI context, quantifying how strongly the signals measured under two different task conditions differ in relation to variance (i.e., *r* ^2^ represents the fraction of the total signal variance which can be explained by the task condition [33]). From a computational point of view, *r* ^2^ simply is the square value of the correlation coefficient between the powers extracted from the EEG signal in the 2 imagery tasks and a fictitious independent variable which assumes 1 of 2 possible different values (e.g., “+1” and “−1”), depending on the imagery task [33].

First of all, for every participant and session we extracted the power spectral densities of signal portions using the modified periodogram (Blackman-Harris window). Secondly, we evaluated all signal powers in the range 8–30 Hz, using 2 Hz-large frequency bins. Finally, *r* ^2^ value was determined for each power bin. For each participant and session, we therefore obtained several *r* ^2^ values which can be grouped according to 3 factors:

1. “threshold,” which has 2 levels corresponding to the conditions “correctly classified and above threshold” and “correctly classified irrespective of threshold,”
2. “channel,” which has 11 levels corresponding to the 11 acquired channels,
3. “frequency,” which has 11 levels corresponding to the 11 2 Hz-large frequency bins in the range 8–30 Hz.

Using a multifactorial statistical test (three-way ANOVA) and multiple comparison tests, we compared the distribution of *r* ^2^ of the “correctly classified and above threshold” and “correctly classified but independent from threshold” signal portions. Secondarily, we also evaluated the effects of the factors “channel” and “frequency.” The obtained *r* ^2^ values were also compiled for each frequency bin in topographical maps of the scalp. Some examples of these maps are shown in Section 3.

## 3. Results

### 3.1. Accuracy

**Figure 5:**
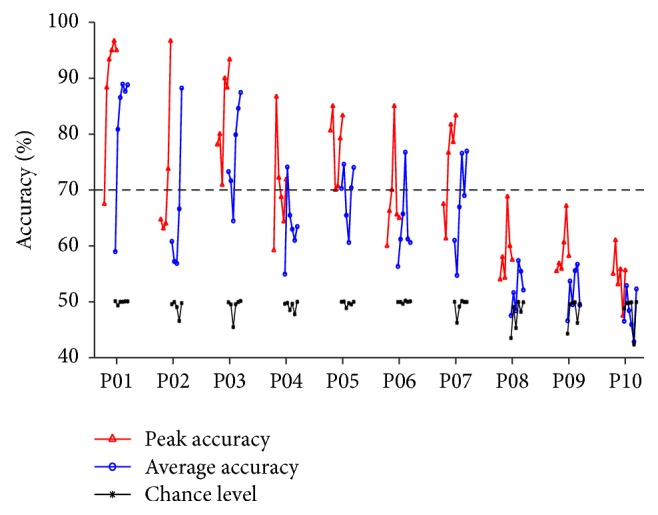
This picture shows the trends over the 6 sessions for peak accuracy (red), average accuracy (blue), and chance level *p*_0_ (black) for each participant. As described in Section 2.4, all the accuracies were computed considering the *U* repetitions following the last *T*of the session. Since each average accuracy should be compared to the corresponding *p*_0_, the blue line of the average and the black line of chance level are aligned. A black horizontal dashed lined indicating the criterion level of 70% is also added.

The detailed results of peak accuracy, average accuracy, and chance level of each participant and session are provided in Table 2 (see Appendix), while Figure 5 shows an overview of the trends of these parameters over the 6 sessions. Additionally, Table S1 in the Supplementary Material available online at http://dx.doi.org/10.1155/2016/4562601 reports the detailed compositions of each training session. The results of the sixth session of P02 are not reported because of artefacts in the respective EEG recordings.

Figure 5 shows that 7 out of 10 participants reached the criterion level of 70% not only with peak but also with average accuracy. In fact, all these participants accessed *C* phase at least once, confirming the average accuracy shown in *U* (Table 2). Moreover, participants P01–P03 reached peak accuracies over 90% in their last session. On the other hand, participants P08–P10 did not reach the criterion level of 70% throughout the 6 sessions, but at least in P08 and P09 the average classification accuracy was significantly different from chance most of the time.

Despite the results of P08–P10, all the participants increased their performance (considering both peak and average accuracy) between the first and last sessions, and the increase was statistically significant over the whole group (*p* < 0.01).

### 3.2. Time Effect

**Figure 6:**
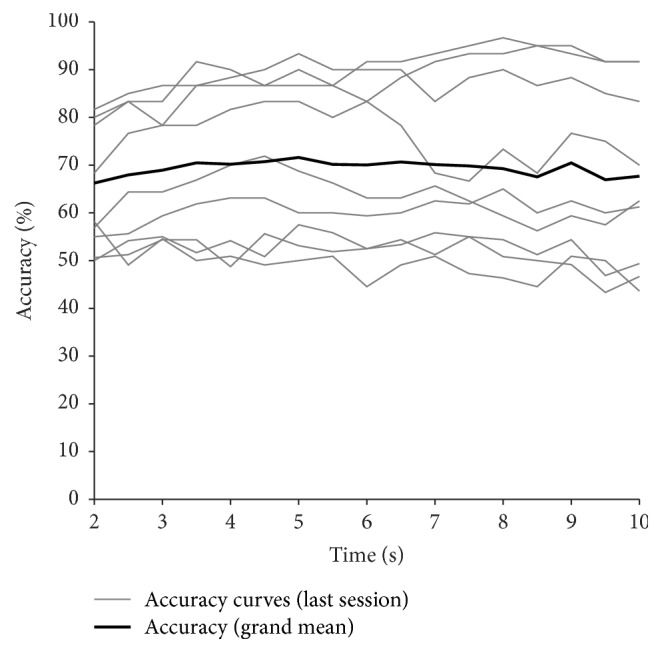
Overview of the trial average accuracy curves for the last session in all participants. Each grey line represents the average accuracy curve of a participant, while the black bold line is computed as the grand mean of the accuracy curves of all participants.

Figure 6 displays the trial average accuracy curves of all participants in their last session. The figure shows that the classifier is not optimized for any specific time-segment; indeed all time-points are generally classified equally well.

### 3.3. Efficacy of the Adaptive Thresholds

**Figure 7:**
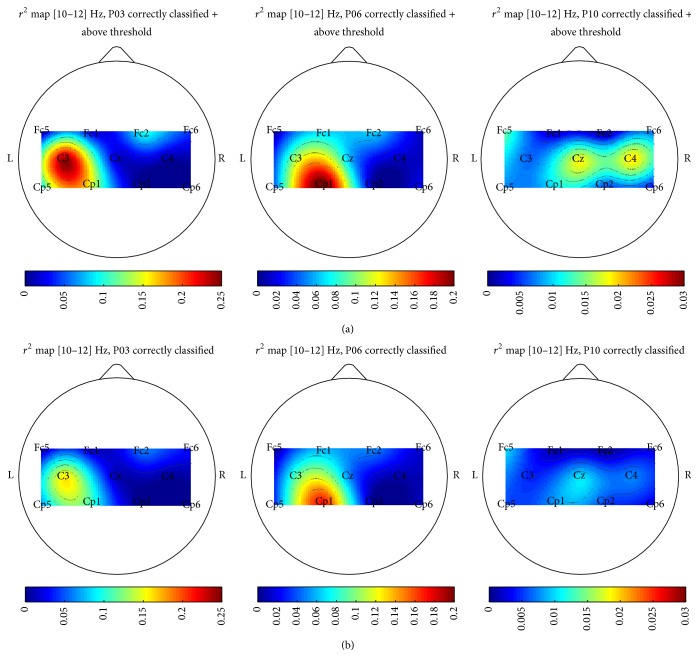
Three examples of *r* ^2^ maps in subject-specific frequency bins. We chose to display 3 participants who exhibited very different levels of BCI control: P03, P06, and P10. The figure shows how *r* ^2^ maps resulting from “correctly classified + above threshold” signals (a) for each participant present approximately the same shape but higher values than the case of “correctly classified” signals without threshold (b). To allow for comparability, the corresponding maps in (a)/(b) share the same colourmap bounds.

The three-way ANOVA revealed that *r* ^2^ values are significantly (*p* < 0.01) higher considering the “correctly classified and above threshold” signals, when compared to generically “correctly classified” signals.  Figure 7 shows several examples of *r* ^2^ topographical maps in the 2 conditions, for 3 different participants and subject-specific bands.

The three-way ANOVA also revealed significant (*p* < 0.01) effects for the factors “frequency” and “channel.” Specifically, it emerged that the bins 8–10 Hz, 10–12 Hz, and 12–14 Hz had significantly higher *r* ^2^ values for the factor “frequency.”

## 4. Discussion

### 4.1. Accuracy

The results showed a significant (*p* < 0.01) increase in performance (in both peak and average accuracy) between the first and the last sessions over the whole set of participants. Seven out of 10 participants reached the criterion level of 70% with both peak and average accuracy, and 3 of them (P01–P03) even obtained >90% peak accuracy in their last session. Three out of 10 users did not manage to reach the criterion level throughout the 6 sessions. However, this result is in line with the well-known phenomenon of BCI illiteracy; that is, BCI control does not work for a nonnegligible portion of users (estimated in 15% to 30%) [20].

The problem of reducing BCI illiteracy was previously investigated by Vidaurre et al. [14, 15], who proposed a multistep adaptive calibration procedure with a high coverage setup (48 electrodes). Because of the large difference in the number of electrodes used, our system is not directly comparable with theirs. However, it could represent a possible portable alternative once the “illiterate” user has gained enough control with a higher coverage approach. Potentially, instead of using a fixed set of 11 electrodes to discriminate the 2 mental states, the number of electrodes could be reduced starting from a high coverage setup using the CSP-based method proposed by Wang et al. [27] or using a procedure based on statistical comparison of the electrode-band power in the two tasks, as described in the work of Mangia et al. [34]. Once the most discriminant subject-specific bands/locations are identified, the number of electrodes could be reduced and a system similar to ours could be used.

As regards the other mentioned studies by Vidaurre et al. and Faller et al. [5, 7, 8] and the study from Xia et al. [12], we can say that the obtained online accuracies are in line with the previously reported measurements.

### 4.2. Time Effect

The results shown in Figure 6 demonstrate the ability of the system to classify the EEG data in each time-point equally well and not only in a short and limited time window. The result is enhanced by the longer duration of our trial with respect to other studies [5, 7–9]. This property makes the system suitable for continuous work as in a real condition of use.

### 4.3. Efficacy of the Adaptive Thresholds

The computed *r* ^2^ values proved to be significantly (*p* < 10^−10^) higher in the case of “correctly classified and above threshold” signals with respect to the simply “correctly classified” signals, and the result is enhanced by the fact that the “correctly classified” signals include the “correctly classified and above threshold” signals. This outcome suggests that the adaptive thresholds we included in the system were actually useful to give feedback only on the most reliable and clear mental states the participant could produce. Moreover, this feature seems to be independent from the participant's level of control. Indeed, looking at Figure 7 showing 3 examples of *r* ^2^ maps choosing 3 participants with widely varying levels of control, we can see how in all cases *r* ^2^ maps resulting from “correctly classified + above threshold” signals present approximately the same shape as the “correctly classified” signals without threshold, but with higher values. We think that this result is particularly interesting considering P10: even if it emerged that this participant could not control the system, the adaptive thresholds still allowed P10 to select the “best” signals possible to give feedback on. Also, considering the shape and frequency bin of *r* ^2^ map, we think it is reasonable to say that it still resembles the physiological ERD/ERS MI patterns, but with weaker *r* ^2^ values than, for example, P03 and P06 (Figure 7). Considering the way we computed the maps, these *r* ^2^ topographies highlight the most different frequency bins/channels between the conditions of right versus left hand MI.

### 4.4. Possible Improvements

Specifically regarding our adaptive algorithm, stability against artefacts is a point that should be improved. In particular, due to the sample covariance (nonrobust covariance matrix estimator),** W** matrix is rather susceptible to artefacts [35]. Our study tried to enhance the system's stability against artefacts in 2 ways: (i) applying a CAR spatial filter in the preprocessing step and (ii) updating** W** using only some selected signals (the “clearest” ones, according to the classifier). Furthermore, since** W** is evaluated by averaging an increasing number of covariance matrices, the influence of a possible unremoved artefact should be gradually reduced. Even so, our system does not assure complete protection against artefacts, and a particularly unlucky *U* session could temporarily lead to confusing feedback.

A real-time artefact recognition algorithm (e.g., the ones proposed in [10, 36, 37]) could be used to further exclude artefacts. This would improve the stability of the** W** matrix and the quality of the training set, avoiding misleading feedbacks and improving the system's accuracy to recognize the classes.

Another simple improvement that should be attempted regards the type of feedback. Since some nonstationarities in EEG come up as reactions to negative feedbacks [38], only correct feedback could be displayed to motivate the participants as much as possible [39].

### 4.5. Overall Comments

Taking the above results and discussions together, we can say that the presented adaptive strategy yields results in line with previously reported findings [5, 7–9]. However, beyond the proposed adaptive strategy, the main novelties presented in this paper regard the way the machine interacts with the user during training.

As regards giving feedback only in case the pattern exceeded an adaptive threshold, the computed *r* ^2^ maps and the 3 examples displayed suggest not only that the threshold effectively selected the best possible patterns to give feedback on but also that these adaptive thresholds work well in users with varying levels of control (notably, this is important with the most critical participants, e.g., P10). Therefore, the inclusion of adaptive thresholds even in systems different from the one introduced here could help to keep challenging the users, irrespective of their ability.

The second idea presented here regards the unbalancing in the presentation of training conditions, in favour of the currently hardest to predict. We suggest that it is reasonable that this feature could improve user training. Indeed, especially when there are two opposing conditions (e.g., “right hand” versus “left hand” or “hand” versus “feet,” whereas this is not the case of, e.g., “hand” versus “rest”), the user may feel more comfortable in one of the conditions, and inherently the system better recognizes it. Insisting on the most misclassified task should theoretically improve the training process, irrespective of the underlying adaptive algorithm.

Finally, we suggest that the last novelty of the described system relies on the concept of “flexible training session.” Indeed, especially with novice users, training may require some trial-and-error before coming up with a good imagination strategy (e.g., tapping a finger, playing an instrument, and brushing teeth). In these cases, if the classifier keeps performing at chance level for too long, it may be pertinent to discard all the acquired data and restart training from the beginning, thereby preventing user discouragement and giving them the chance to try a different imagination strategy. On the other hand, as users reach a satisfactory level of control, the training phase should not be needlessly long to avoid annoying them. In addition, it is desirable that these “decisions” are taken automatically by the system. Considering all these elements, we think it is the modular structure that makes the system so flexible. To give a further example, specifically in the case of our system, if *C* phase is accessed too early, nothing stops *U* repetition being inserted to tune the system's parameters quickly and catch up with the user's evolution. We suggest that a modular structure, similar to the one introduced here, could help build maximally flexible and customizable BCI systems.

## 5. Conclusion

The present study developed a fully automate plug-and-play BCI system to control the flexion-extension of a 1 DOF-modelled arm using MI strategy. The system was tested online on 10 participants, of whom 7 reached the criterion level of 70% with both peak and average accuracy in just 3 days. Despite these results, the system still presents the major limitation of not being completely robust against EEG artefacts. In particular, in the considered frequency band (8–30 Hz), muscular artefacts are the most critical. The inclusion of an artefact recognition algorithm should theoretically further improve the system's stability and the quality of the feedback. Another simple improvement to the system could stem from the decision to display only correct feedback to prevent nonstationarities which come up as reactions to frustrating feedback and motivate the participants as much as possible.

The presented system falls within the category of adaptive systems that aim to improve the user training procedure, dealing with the nonstationarities elicited by feedback training. Beyond the proposed adaptive strategy, we suggest that the main ideas/novelties introduced in the system are to (i) keep challenging the user through the use of adaptive thresholds in the feedback phase, (ii) present imbalanced training conditions, insisting on the most difficult one for the user, and (iii) adapt the type and length of the training session, depending on user performances.

## Supplementary Material

The Supplementary Material show the detailed composition, in terms of sequences of *T*, *U* and *C* modules, for each participant and session.

## Figures and Tables

**Table 1:** Overview of the module characteristics.

*T*	*U*	*C*
(i) One *T* repetition	(i) Several *U* repetitions	(i) Several *C* repetitions
(ii) MI instruction via arrow presentation	(ii) MI instruction via arrow presentation	(ii) MI instruction via target position
(iii) No feedback	(iii) Feedback (above threshold)	(iii) Feedback (above threshold)
	(iv) Adaptively updated thresholds	(iv) Thresholds no longer updated
(iv) First estimation of **W** and SVM classifier	(v) Use of **W** and SVM and its updating	(v) Use of **W** and SVM
(v) Balanced arrows	(vi) Imbalanced arrows	
	(vii) Accuracy evaluation	(vi) Accuracy evaluation

**Table 2:** Detailed results for each participant and session. The first 2 columns list the name of the participant and the number of sessions. The third, fourth, and fifth columns show, respectively, the peak accuracy (%), average accuracy (%), and chance level (%) obtained considering the *U* repetitions following the last *T* of each session. The sixth column shows the result of the comparison via confidence intervals (*α* = 0.05) between the average accuracy and *p*_0_ (yes = the average accuracy is significantly different from chance; no = otherwise). The seventh column finally refers to the ITR (bits/min) of the corresponding session. In case *C* phase was reached, the last 4 columns show, respectively, the average accuracy (%), the chance level *p*_0_ (%), the result of comparison between the two (*α* = 0.05), and the ITR (bits/min) considering the three repetitions of *C*.

Participant	*S*	*U* (peak) %	*U* (mean) %	*p*_0_ (chance) %	*α* (0.05)	ITR (bits/min)	*C* (mean) %	*p*_0_ (chance) %	*α* (0.05)	ITR (bits/min)
P01	1	67.50	58.89	50.14	Yes	2.76	—	—	—	—
	2	88.33	80.88	49.34	Yes	35.53	89.10	51.72	Yes	60.37
	3	93.33	86.57	50.00	Yes	51.70	90.74	61.65	Yes	66.59
	4	95.00	88.92	50.00	Yes	59.72	84.95	58.91	Yes	46.67
	5	96.67	87.65	50.08	Yes	55.27	96.53	51.99	Yes	93.91
	6	95.00	88.82	50.10	Yes	59.37	91.08	49.73	Yes	67.95

P02	1	64.71	60.83	49.56	Yes	4.09	—	—	—	—
	2	63.13	57.21	49.95	Yes	1.80	—	—	—	—
	3	64.00	56.88	49.08	Yes	1.64	—	—	—	—
	4	73.75	66.62	46.56	Yes	9.75	66.46	57.35	Yes	9.56
	5	96.67	88.24	49.78	Yes	57.29	92.22	54.78	Yes	72.67

P03	1	78.13	73.31	49.94	Yes	19.56	83.46	54.71	Yes	42.35
	2	80.00	71.67	49.53	Yes	16.81	58.78	48.91	Yes	2.68
	3	70.91	64.49	45.49	Yes	7.38	87.18	58.77	Yes	53.70
	4	90.00	79.90	49.57	Yes	33.13	81.91	54.70	Yes	38.16
	5	88.33	84.61	49.98	Yes	45.65	77.09	54.26	Yes	26.83
	6	93.33	87.45	50.16	Yes	54.61	82.53	51.51	Yes	39.80

P04	1	59.23	54.98	49.66	Yes	0.86	—	—	—	—
	2	86.67	74.12	49.82	Yes	21.0	74.82	54.54	Yes	22.31
	3	72.22	65.49	48.52	Yes	8.45	60.20	48.75	Yes	3.63
	4	68.75	62.98	49.66	Yes	5.90	—	—	—	—
	5	64.38	60.99	47.80	Yes	4.22	—	—	—	—
	6	71.88	63.46	50.01	Yes	6.35	—	—	—	—

P05	1	80.63	70.29	50.00	Yes	14.68	68.01	49.83	Yes	11.49
	2	85.00	74.61	50.04	Yes	21.91	73.34	55.29	Yes	19.62
	3	70.00	65.49	48.89	Yes	8.45	50.45	45.24	Yes	0.01
	4	70.63	60.62	49.76	Yes	3.94	—	—	—	—
	5	79.23	70.41	49.54	Yes	14.85	66.13	48.81	Yes	9.17
	6	83.33	74.02	50.01	Yes	20.83	74.22	49.05	Yes	21.19

P06	1	60.00	56.32	49.95	Yes	1.39	—	—	—	—
	2	66.25	61.21	49.98	Yes	4.39	—	—	—	—
	3	70.00	65.74	49.66	Yes	8.72	47.09	39.73	Yes	0.29
	4	85.00	76.76	50.21	Yes	26.15	80.00	51.04	Yes	33.37
	5	65.63	61.25	50.00	Yes	4.42	—	—	—	—
	6	65.00	60.62	50.07	Yes	3.94	—	—	—	—

P07	1	67.50	60.99	50.00	Yes	4.22	—	—	—	—
	2	61.33	54.75	46.26	Yes	0.78	—	—	—	—
	3	76.67	66.96	49.16	Yes	10.16	50.94	42.90	Yes	0.03
	4	81.67	76.57	50.16	Yes	25.75	73.44	51.23	Yes	19.79
	5	78.57	68.99	49.96	Yes	12.81	58.49	46.54	Yes	2.51
	6	83.33	76.96	49.95	Yes	26.56	61.15	47.57	Yes	4.34

P08	1	54.00	47.53	43.53	Yes	0.21	—	—	—	—
	2	58.00	51.65	49.07	No	0.09	—	—	—	—
	3	54.29	48.40	45.33	Yes	0.09	—	—	—	—
	4	68.82	57.37	49.96	Yes	1.89	—	—	—	—
	5	60.00	55.48	48.23	Yes	1.04	—	—	—	—
	6	57.50	52.11	49.93	Yes	0.15	—	—	—	—

P09	1	55.45	46.63	44.30	Yes	0.39	—	—	—	—
	2	56.88	53.71	49.58	Yes	0.48	—	—	—	—
	3	55.88	49.48	49.84	No	0.01	—	—	—	—
	4	60.62	55.59	49.94	Yes	1.08	—	—	—	—
	5	67.14	56.72	46.21	Yes	1.57	—	—	—	—
	6	58.18	49.41	49.66	No	0.01	—	—	—	—

P10	1	55.00	46.54	48.85	No	0.41	—	—	—	—
	2	61.00	52.88	49.79	Yes	0.29	—	—	—	—
	3	53.13	48.46	49.80	No	0.08	—	—	—	—
	4	55.83	45.93	49.92	No	0.57	—	—	—	—
	5	47.50	42.87	42.37	No	1.77	—	—	—	—
	6	55.63	52.32	49.98	Yes	0.19	—	—	—	—

## Appendix

See Table 2.
