# Supplementary material for: EEG-Based BCI System Using Adaptive Features Extraction and Classification Procedures
Source: Comput Intell Neurosci. 2016 Aug 17;2016:4562601. doi: 10.1155/2016/4562601 (PMC5011245; doi:10.1155/2016/4562601)
Supplement: Supplementary file 1 — The Supplementary Material show the detailed composition, in terms of sequences of T, U and C modules, for each participant and session. [file 4562601.f1.pdf]

Supplementary Materials

TABLE S1: This table shows the detailed sequences of *T*, *U* and *C* for each participant and session. As in TABLE 2 and FIGURE 6, the sequences of the 6th session of S02 are not shown because of artifacts in the EEG.

Participant	S	Sequence
P01	1	$T\ U_1\ U_2\ U_3\ U_4\ U_5\ U_6\ U_7\ U_8\ T\ U_9\ U_{10}\ U_{11}\ U_{12}\ U_{13}\ U_{14}\ U_{15}\ U_{16}$
	2	$T\ U_1\ U_2\ U_3\ U_4\ U_5\ U_6\ C_1\ C_2\ C_3$
	3	$T\ U_1\ U_2\ U_3\ U_4\ U_5\ U_6\ C_1\ C_2\ C_3$
	4	$T\ U_1\ U_2\ U_3\ U_4\ U_5\ U_6\ C_1\ C_2\ C_3$
	5	$T\ U_1\ U_2\ U_3\ U_4\ U_5\ U_6\ C_1\ C_2\ C_3$
	6	$T\ U_1\ U_2\ U_3\ U_4\ U_5\ U_6\ C_1\ C_2\ C_3$
P02	1	$T\ U_1\ U_2\ U_3\ U_4\ U_5\ U_6\ U_7\ U_8\ U_9\ U_{10}\ U_{11}\ U_{12}\ U_{13}\ U_{14}\ U_{15}\ U_{16}$
	2	$T\ U_1\ U_2\ U_3\ U_4\ U_5\ U_6\ U_7\ U_8\ U_9\ U_{10}\ U_{11}\ U_{12}\ U_{13}\ U_{14}\ U_{15}\ U_{16}$
	3	$T\ U_1\ U_2\ U_3\ U_4\ U_5\ U_6\ T\ U_7\ U_8\ U_9\ U_{10}\ U_{11}\ U_{12}\ U_{13}\ U_{14}\ U_{15}\ U_{16}$
	4	$T\ U_1\ U_2\ U_3\ U_4\ U_5\ U_6\ U_7\ U_8\ T\ U_9\ U_{10}\ U_{11}\ U_{12}\ U_{13}\ U_{14}\ U_{15}\ U_{16}\ C_1\ C_2\ C_3$
	5	$T\ U_1\ U_2\ U_3\ U_4\ U_5\ U_6\ C_1\ C_2\ C_3$
P03	1	$T\ U_1\ U_2\ U_3\ U_4\ U_5\ U_6\ U_7\ U_8\ U_9\ U_{10}\ U_{11}\ U_{12}\ U_{13}\ U_{14}\ U_{15}\ U_{16}\ C_1\ C_2\ C_3$
	2	$T\ U_1\ U_2\ U_3\ U_4\ U_5\ U_6\ C_1\ C_2\ C_3$
	3	$T\ U_1\ U_2\ U_3\ U_4\ U_5\ U_6\ U_7\ U_8\ U_9\ U_{10}\ U_{11}\ C_1\ C_2\ C_3$
	4	$T\ U_1\ U_2\ U_3\ U_4\ U_5\ U_6\ C_1\ C_2\ C_3$
	5	$T\ U_1\ U_2\ U_3\ U_4\ U_5\ U_6\ C_1\ C_2\ C_3$
	6	$T\ U_1\ U_2\ U_3\ U_4\ U_5\ U_6\ C_1\ C_2\ C_3$
P04	1	$T\ U_1\ U_2\ U_3\ T\ U_4\ U_5\ U_6\ U_7\ U_8\ U_9\ U_{10}\ U_{11}\ U_{12}\ U_{13}\ U_{14}\ U_{15}\ U_{16}$
	2	$T\ U_1\ U_2\ U_3\ U_4\ U_5\ U_6\ C_1\ C_2\ C_3$
	3	$T\ U_1\ U_2\ U_3\ U_4\ U_5\ U_6\ U_7\ U_8\ U_9\ C_1\ C_2\ C_3$
	4	$T\ U_1\ U_2\ U_3\ U_4\ U_5\ U_6\ U_7\ U_8\ U_9\ U_{10}\ U_{11}\ U_{12}\ U_{13}\ U_{14}\ U_{15}\ U_{16}$
	5	$T\ U_1\ U_2\ U_3\ U_4\ U_5\ U_6\ U_7\ U_8\ U_9\ U_{10}\ U_{11}\ U_{12}\ U_{13}\ U_{14}\ U_{15}\ U_{16}$
	6	$T\ U_1\ U_2\ U_3\ U_4\ U_5\ U_6\ U_7\ U_8\ U_9\ U_{10}\ U_{11}\ U_{12}\ U_{13}\ U_{14}\ U_{15}\ U_{16}$
P05	1	$T\ U_1\ U_2\ U_3\ U_4\ U_5\ U_6\ U_7\ U_8\ U_9\ U_{10}\ U_{11}\ U_{12}\ U_{13}\ U_{14}\ U_{15}\ U_{16}\ C_1\ C_2\ C_3$
	2	$T\ U_1\ U_2\ U_3\ U_4\ U_5\ U_6\ C_1\ C_2\ C_3$
	3	$T\ U_1\ U_2\ U_3\ T\ U_4\ U_5\ U_6\ U_7\ U_8\ U_9\ U_{10}\ U_{11}\ U_{12}\ U_{13}\ U_{14}\ U_{15}\ U_{16}\ C_1\ C_2\ C_3$
	4	$T\ U_1\ U_2\ U_3\ U_4\ U_5\ U_6\ U_7\ U_8\ U_9\ U_{10}\ U_{11}\ U_{12}\ U_{13}\ U_{14}\ U_{15}\ U_{16}$
	5	$T\ U_1\ U_2\ U_3\ U_4\ U_5\ U_6\ U_7\ U_8\ U_9\ U_{10}\ U_{11}\ U_{12}\ U_{13}\ C_1\ C_2\ C_3$
	6	$T\ U_1\ U_2\ U_3\ U_4\ U_5\ U_6\ C_1\ C_2\ C_3$
P06	1	$T\ U_1\ U_2\ U_3\ U_4\ U_5\ U_6\ U_7\ U_8\ U_9\ U_{10}\ U_{11}\ U_{12}\ U_{13}\ U_{14}\ U_{15}\ U_{16}$
	2	$T\ U_1\ U_2\ U_3\ U_4\ U_5\ U_6\ U_7\ U_8\ U_9\ U_{10}\ U_{11}\ U_{12}\ U_{13}\ U_{14}\ U_{15}\ U_{16}$
	3	$T\ U_1\ U_2\ U_3\ U_4\ U_5\ U_6\ U_7\ U_8\ U_9\ U_{10}\ U_{11}\ U_{12}\ U_{13}\ U_{14}\ U_{15}\ U_{16}\ C_1\ C_2\ C_3$
	4	$T\ U_1\ U_2\ U_3\ U_4\ U_5\ U_6\ C_1\ C_2\ C_3$
	5	$T\ U_1\ U_2\ U_3\ U_4\ U_5\ U_6\ U_7\ U_8\ U_9\ U_{10}\ U_{11}\ U_{12}\ U_{13}\ U_{14}\ U_{15}\ U_{16}$
	6	$T\ U_1\ U_2\ U_3\ U_4\ U_5\ U_6\ U_7\ U_8\ U_9\ U_{10}\ U_{11}\ U_{12}\ U_{13}\ U_{14}\ U_{15}\ U_{16}$
P07	1	$T\ U_1\ U_2\ U_3\ U_4\ U_5\ U_6\ U_7\ U_8\ U_9\ U_{10}\ U_{11}\ U_{12}\ U_{13}\ U_{14}\ U_{15}\ U_{16}$
	2	$T\ U_1\ U_2\ U_3\ U_4\ U_5\ U_6\ U_7\ U_8\ U_9\ U_{10}\ U_{11}\ U_{12}\ U_{13}\ U_{14}\ U_{15}\ U_{16}$
	3	$T\ U_1\ U_2\ U_3\ U_4\ U_5\ U_6\ U_7\ U_8\ U_9\ U_{10}\ U_{11}\ U_{12}\ C_1\ C_2\ C_3$
	4	$T\ U_1\ U_2\ U_3\ U_4\ U_5\ U_6\ C_1\ C_2\ C_3$
	5	$T\ U_1\ U_2\ U_3\ U_4\ U_5\ U_6\ U_7\ C_1\ C_2\ C_3$
	6	$T\ U_1\ U_2\ U_3\ U_4\ U_5\ U_6\ C_1\ C_2\ C_3$

P08	1	$T\ U_1\ U_2\ U_3\ U_4\ U_5\ U_6\ U_7\ U_8\ U_9\ U_{10}\ U_{11}\ T\ U_{12}\ U_{13}\ U_{14}\ U_{15}\ U_{16}$
	2	$T\ U_1\ U_2\ U_3\ T\ U_4\ U_5\ U_6\ U_7\ U_8\ U_9\ U_{10}\ U_{11}\ T\ U_{12}\ U_{13}\ U_{14}\ U_{15}\ U_{16}$
	3	$T\ U_1\ U_2\ U_3\ U_4\ U_5\ U_6\ U_7\ U_8\ T\ U_9\ U_{10}\ U_{11}\ U_{12}\ U_{13}\ U_{14}\ U_{15}\ U_{16}$
	4	$T\ U_1\ U_2\ U_3\ U_4\ U_5\ U_6\ U_7\ U_8\ U_9\ U_{10}\ U_{11}\ U_{12}\ U_{13}\ U_{14}\ U_{15}\ U_{16}$
	5	$T\ U_1\ U_2\ U_3\ U_4\ U_5\ U_6\ U_7\ U_8\ U_9\ U_{10}\ U_{11}\ U_{12}\ U_{13}\ U_{14}\ U_{15}\ U_{16}$
	6	$T\ U_1\ U_2\ U_3\ U_4\ T\ U_5\ U_6\ U_7\ U_8\ U_9\ U_{10}\ U_{11}\ U_{12}\ U_{13}\ U_{14}\ U_{15}\ U_{16}$
P09	1	$T\ U_1\ U_2\ U_3\ U_4\ U_5\ T\ U_6\ U_7\ U_8\ U_9\ U_{10}\ U_{11}\ U_{12}\ U_{13}\ U_{14}\ U_{15}\ U_{16}$
	2	$T\ U_1\ U_2\ U_3\ U_4\ U_5\ U_6\ U_7\ U_8\ U_9\ U_{10}\ U_{11}\ U_{12}\ U_{13}\ U_{14}\ U_{15}\ U_{16}$
	3	$T\ U_1\ U_2\ U_3\ U_4\ U_5\ U_6\ U_7\ U_8\ U_9\ U_{10}\ U_{11}\ U_{12}\ U_{13}\ U_{14}\ U_{15}\ U_{16}$
	4	$T\ U_1\ U_2\ U_3\ U_4\ U_5\ U_6\ U_7\ U_8\ U_9\ U_{10}\ U_{11}\ U_{12}\ U_{13}\ U_{14}\ U_{15}\ U_{16}$
	5	$T\ U_1\ U_2\ U_3\ U_4\ U_5\ U_6\ U_7\ U_8\ U_9\ T\ U_{10}\ U_{11}\ U_{12}\ U_{13}\ U_{14}\ U_{15}\ U_{16}$
	6	$T\ U_1\ U_2\ U_3\ U_4\ U_5\ U_6\ T\ U_7\ U_8\ U_9\ U_{10}\ U_{11}\ U_{12}\ U_{13}\ U_{14}\ U_{15}\ U_{16}$
P10	1	$T\ U_1\ U_2\ U_3\ U_4\ U_5\ U_6\ U_7\ U_8\ U_9\ U_{10}\ U_{11}\ U_{12}\ U_{13}\ U_{14}\ U_{15}\ U_{16}$
	2	$T\ U_1\ U_2\ U_3\ T\ U_4\ U_5\ U_6\ T\ U_7\ U_8\ U_9\ U_{10}\ U_{11}\ U_{12}\ U_{13}\ U_{14}\ U_{15}\ U_{16}$
	3	$T\ U_1\ U_2\ U_3\ U_4\ U_5\ U_6\ U_7\ U_8\ U_9\ U_{10}\ U_{11}\ U_{12}\ U_{13}\ U_{14}\ U_{15}\ U_{16}$
	4	$T\ U_1\ U_2\ U_3\ U_4\ T\ U_5\ U_6\ U_7\ U_8\ U_9\ U_{10}\ U_{11}\ U_{12}\ U_{13}\ U_{14}\ U_{15}\ U_{16}$
	5	$T\ U_1\ U_2\ U_3\ U_4\ T\ U_5\ U_6\ U_7\ U_8\ T\ U_9\ U_{10}\ U_{11}\ U_{12}\ U_{13}\ U_{14}\ U_{15}\ U_{16}$
	6	$T\ U_1\ U_2\ U_3\ U_4\ U_5\ U_6\ U_7\ U_8\ U_9\ U_{10}\ U_{11}\ U_{12}\ U_{13}\ U_{14}\ U_{15}\ U_{16}$
